# Editorial: Oxidative stress in degenerative bone and joint diseases: novel molecular mechanisms and therapeutic potential

**DOI:** 10.3389/fmolb.2023.1213380

**Published:** 2023-05-30

**Authors:** Jialiang Lin, Libo Jiang, Ji Tu, Xiangyang Wang, Weishi Li

**Affiliations:** ^1^ Department of Orthopaedics, Peking University Third Hospital, Peking University, Beijing, China; ^2^ Engineering Research Center of Bone and Joint Precision Medicine, Ministry of Education, Beijing, China; ^3^ Department of Orthopaedic Surgery, Zhongshan Hospital, Fudan University, Shanghai, China; ^4^ Spine Labs, St. George and Sutherland Clinical School, Faculty of Medicine, University of New South Wales, Sydney, NSW, Australia; ^5^ Department of Orthopaedics, The Second Affiliated Hospital and Yuying Children’s Hospital of Wenzhou Medical University, Wenzhou, China

**Keywords:** oxidative stress, degenerative bone and joint diseases, cell death, senescence, therapeutic targets, diagnostic biomarkers

Degenerative bone and joint diseases (DBJDs) are one of the major causes of health problems affecting the middle-aged and elderly population, imposing a huge medical burden on both individuals and society (Imagama et al., 2020). For example, approximately 10% of noninstitutionalized adults in the United States are afflicted with osteoarthritis (OA), resulting in an estimated $45 billion in excess annual medical costs nationwide (Zhao et al., 2019). Moderate oxidative stress is a protective mechanism for cells in response to various stresses and plays an important role in maintaining cellular homeostasis. However, oxidative stress damage occurs when cellular antioxidant defenses are overwhelmed by excessive generation of free radicals such as reactive oxygen species (ROS) and reactive nitrogen species (RNS), which involve almost all components of the cell, including DNA, proteins, and lipids. Currently, oxidative stress has been shown to be associated with the onset and progression of DBJDs such as OA, intervertebral disc degeneration (IDD), and osteoporosis (Lin et al., 2020; Coryell et al., 2021; Kimball et al., 2021). Specifically, oxidative stress plays an essential role in the senescence and death of cells associated with DBJDs (Lin et al., 2020; Coryell et al., 2021). The Research Topic of “Oxidative Stress in Degenerative Bone and Joint Diseases: Novel Molecular Mechanisms and Therapeutic Potential” investigates the potential molecular mechanisms of oxidative stress in DBJDs and explores novel diagnostic approaches and therapeutic strategies with regard to oxidative stress as a target.

Cellular senescence is one of the major pathological manifestations of DBJDs. Numerous pieces of evidence suggest that oxidative stress-induced damage plays an important role in the cellular senescence process (Finkel and Holbrook, 2000; Erusalimsky, 2020). First, telomeres are extremely sensitive to oxidative damage, yet they are far less capable of repair than other parts of the chromosome (von Zglinicki, 2000). As a result, oxidative damage may lead to telomere attrition, which accelerates cellular senescence and increases the risk of age-related diseases (Blackburn et al., 2015; Erusalimsky, 2020). In addition, abnormally high levels of oxidative stress cause a range of cellular phenotypic alterations, including altered expression of senescence-associated genes and arrest of the cellular proliferation cycle (Hernandez-Segura et al., 2018). Notably, oxidative damage is the result of the breakdown of cellular antioxidant defenses by excessive free radicals, and the effectiveness of antioxidant supplementation in ameliorating cellular senescence and organ degeneration demonstrates another perspective on the importance of redox homeostasis in arresting cellular senescence (de Picciotto et al., 2016; Chang et al., 2022). Wen et al. reviewed the link between oxidative stress, cellular senescence, and degenerative diseases in their article. Stress-induced premature senescence and natural senescence of cells can cause mitochondrial dysfunction and lead to substantial ROS generation. In turn, excessive ROS contribute to cellular oxidative damage and accelerate senescence, resulting in the further development of age-related degenerative diseases.

Aberrant cell death is another major feature of DBJDs. With a deeper understanding of cell death types, the existence of apoptosis, necroptosis, pyroptosis, and ferroptosis in DBJDs has been gradually confirmed (Yang et al., 2021). Oxidative stress, as an important adaptive mechanism for cell survival, is inevitably involved in the process of these different types of cell death. Apoptosis induced by oxidative stress, as a classical mode of cell death, has been confirmed by numerous studies for its important contribution to bone and joint degeneration (Ding et al., 2013; Hosseinzadeh et al., 2016). Notably, ferroptosis, which is also closely related to oxidative stress, has received increasing attention in recent years for its role in DBJDs. Ferroptosis is an iron-dependent programmed cell death characterized by lipid peroxidation. It is generally thought to be triggered by dysfunction of the antioxidant system of glutathione accompanied by overproduction of ROS, leading to uncontrolled lipid peroxidation and eventual cell death (Yang and Stockwell, 2016). In recent years, new evidence continues to emerge suggesting that ferroptosis plays an important role in the pathogenesis of DBJDs such as IDD and OA (Zhang et al., 2021; Zhang et al., 2022). In this Research Topic, Xia et al. found ferroptosis-related genes (FRGs) that may be involved in OA synovitis through bioinformatics analysis. Further, they identified seven hub genes (ATF3, IL6, CDKN1A, IL1B, EGR1, JUN, and CD44) among these FRGs and showed by ROC analysis that almost all these hub genes have good diagnostic properties. This study suggests that these hub genes are promising diagnostic biomarkers and therapeutic targets for synovitis during OA and provide additional bioinformatics at the transcriptome level to reveal the pathogenesis of OA. In addition, Wang et al. also screened for FRGs differentially expressed in OA by bioinformatics analysis and validated their biological functions in chondrocytes. The results showed that the expression of ATF3 and TFRC was elevated in interleukin-1β (IL-1β)-stimulated chondrocytes, while the expression of CXCL2 and JUN was decreased. And for the first time, it was found that the upregulation of TFRC expression in IL-1β-stimulated chondrocytes could be reversed by ferroptosis inhibitors. These results suggest that the ferroptosis of chondrocytes may be an important factor in the development of OA, while TFRC may be an accomplice for the process of ferroptosis in IL-1β-stimulated chondrocytes, which is expected to be a potential target for OA therapy in the future. The studies by Xia et al. and Wang et al. both revealed the possible involvement of ferroptosis in the pathogenesis of OA and identified some common differentially expressed FRGs in OA, but their actual role in OA remains to be further validated.

Cheng et al. analyze the scientific output of oxidative stress in IDD from 2007 to 2021. In their study, they found a continuous increase in annual publications in the field since 2013, especially in the last 2 years. To some extent, this indicates that the role of oxidative stress in IDD is receiving increasing attention from researchers. In addition, Wen et al. reviewed the effects of oxidative stress and aging on IDD and provided insight into the treatment of IDD through targeted inhibition of oxidative stress. Liu et al. reviewed the role and mechanisms of oxidative stress in the progression of knee osteoarthritis and summarized current attempts to target oxidative stress in the treatment of OA. It is worth mentioning that all of these studies emphasized the importance of mitochondria in oxidative stress-induced bone and joint degeneration, as it plays a significant role in both the generation and clearance of ROS. This suggests that we should pay more attention to the management of mitochondria in oxidative stress in the future.

In conclusion, this Research Topic provides new insights into the role and mechanisms of oxidative stress in DBJDs and explores potential therapeutic strategies. With a better understanding of the mechanisms regulating redox homeostasis, the importance of some cellular organelles such as mitochondria and proteins has been increasingly emphasized. Hence, in-depth studies targeting mitochondria and their interactive regulatory networks are expected to be an important breakthrough point in the management of oxidative stress in DBJDs. Undeniably, further studies are still needed in the future to realize the benefit of patients with DBJDs through the control of oxidative stress.
